# Supplementary material for: Multiclass classification of Autism Spectrum Disorder, attention deficit hyperactivity disorder, and typically developed individuals using fMRI functional connectivity analysis
Source: PLoS One. 2024 Oct 17;19(10):e0305630. doi: 10.1371/journal.pone.0305630 (PMC11486369; doi:10.1371/journal.pone.0305630)
Supplement: Attachment — Submitted filename: Author_response_to_review_comments_PLOS_ONE_ADHD_ASD (3).pdf [file pone.0305630.s003.pdf]

Authors’ Response to Reviews of

Multiclass machine learning and deep learning for analysis of fMRI functional connectivity from individuals with Autism Spectrum Disorder and Attention Deficit Hyperactivity Disorder

Caroline L. Alves1, Tiago Martinelli, Loriz Francisco Sallum, Francisco Aparecido Rodrigues, Thaise G. L. de O. Toutain, Joel Augusto Moura Porto, Christiane Thielemann, Patricia Maria de Carvalho Aguiar, Michael Moeckel
PLOS ONE,

RC: ***Reviewers’ Comment***, AR: Authors’ Response, ☐ Manuscript Text

We would like to thank you the reviewer for carefully reading our manuscript. The comments have improved the manuscript. We provide point-by-point responses to each comment below.

1. Reviewer 1

The authors used multiclass machine learning and deep learning algorithms to distinguish ASD, ADHD, and TD. The study has a lot of major issues, which I will outline below following the order of the manuscript:

RC: ***The title does not accurately represent their current work. Since the present study was focused on the classification of ASD, ADHD, and TD, there should be more descriptions involving classification.***

AR: We appreciate your insightful comments regarding the Title of our paper. Upon reflection, we agree that the current Title may not accurately convey the primary focus of our study, which indeed centers on the classification of Autism Spectrum Disorder (ASD), Attention Deficit Hyperactivity Disorder (ADHD), and typically developed (TD) individuals using machine learning techniques.

In response to your suggestion, we propose a revised title that explicitly highlights the classification aspect of our research: Revised Title: "Multiclass Classification of Autism Spectrum Disorder, Attention Deficit Hyperactivity Disorder, and Typically Developed Individuals Using fMRI Functional Connectivity Analysis"

We believe this revised Title better encapsulates our study’s core objective and accurately communicates the methodology and findings presented in the manuscript.

RC: ***The Abstract of this manuscript is excessively redundant and lacks a concise summary of the article’s innovation. The authors should try to rewrite this section by following a specific order and to make it more readable for the readers.***

AR: We appreciate your feedback and constructive criticism regarding the abstract of our manuscript. We have carefully considered your suggestions and made significant revisions to improve clarity and conciseness while highlighting the innovation of our study.

Revised Abstract:

"Neurodevelopmental conditions, such as Autism Spectrum Disorder (ASD) and Attention Deficit Hyperactivity Disorder (ADHD), present unique challenges due to overlapping symptoms, making an accurate diagnosis and targeted intervention difficult. Our study employs advanced machine learning techniques to analyze functional magnetic resonance imaging (fMRI) data from individuals with ASD, ADHD, and typically developed (TD) controls, totaling 120 subjects in the study. Leveraging multiclass classification (ML) algorithms, we achieve superior accuracy in distinguishing between ASD, ADHD, and TD groups, surpassing existing benchmarks with an area under the ROC curve near 98%. Our analysis reveals distinct neural signatures associated with ASD and ADHD: individuals with ADHD exhibit altered connectivity patterns of regions involved in attention and impulse control, whereas those with ASD show disruptions in brain regions critical for social and cognitive functions. The observed connectivity patterns, on which the ML classification rests, agree with established diagnostic approaches based on clinical symptoms.

Furthermore, complex network analyses highlight differences in brain network integration and segregation among the three groups. Our findings pave the way for refined, ML-enhanced diagnostics in accordance with established practices, offering a promising avenue for developing trustworthy clinical decision-support systems."

RC: ***The Introduction needs to be roundly restructured so that it clearly reflects the hypothesis, questions, and aim of the study. Besides, in the fifth and seventh paragraphs of the Introduction, necessary contents and explanations are lacking.***

AR: Thank you for your valuable feedback on our manuscript. We have carefully considered your suggestions and substantially revised the Introduction (section 1. Introduction). We aim to provide a more precise delineation of the study's hypothesis, questions, and aims and incorporate the necessary content and explanations lacking in the fifth and seventh paragraphs.

RC: ***It seems that the neuroimaging data of ASD and ADHD were obtained from two independent datasets, how did the authors eliminate the effects between different sites?***

AR: We appreciate your inquiry regarding the potential effects of utilizing neuroimaging data from independent datasets. To address this concern, we employed rigorous preprocessing procedures to harmonize the data and minimize site-related variations.

Specifically, we utilized the NeuroImaging Analysis Kit (NIAK) preprocessing pipeline, which incorporates various steps to standardize and enhance the quality of functional magnetic resonance imaging (fMRI) data. NIAK offers functionalities for motion correction, slice timing correction, spatial normalization, and nuisance signal regression, among others. These preprocessing steps are essential for reducing confounding factors and ensuring data consistency across different acquisition sites. Further, we utilized the same neuroimaging analysis tools and atlases for both datasets. Specifically, we employed the Nilearn package for preprocessing and analysis, which offers standardized methods for handling and analyzing neuroimaging data. We also utilized the Bootstrap Analysis of Stable Clusters (BASC) atlas to define brain regions of interest (ROIs) in both datasets. The BASC atlas provides a standardized parcellation of the brain, ensuring that the same ROIs were used across ASD and ADHD datasets.

Finally, we employed data augmentation techniques such as sliding time window analysis to further address potential side effects and enhance generalization. Using sliding time windows of 20 seconds, we aimed to capture temporal dynamics while minimizing the influence of specific site-related artifacts.

We added the following text to the paper 2.1. Data and data preprocessing section:

"In addition to the aforementioned preprocessing steps, we employed the NeuroImaging Analysis Kit (NIAK) [53] to further standardize and enhance the quality of our neuroimaging data for both datasets. NIAK offers a comprehensive set of tools for preprocessing fMRI data, including motion correction, slice timing correction, spatial normalization, and nuisance signal regression [37, 54]. These preprocessing procedures are crucial for mitigating potential confounding factors introduced by differences in data acquisition protocols across multiple sites. By implementing the same preprocessing pipeline in both datasets, we aimed to minimize site-related variations and ensure the consistency and reliability of our data across different acquisition sites. This standardized approach facilitated the integration of neuroimaging data from disparate sources, enhancing the validity and generalizability of our findings."

Further, we also try to distinguish the TD matrices of these two different datasets obtained after preprocessing. The results depicted in Figure 4. demonstrate that it was not possible to distinguish between these two control groups, proving that we could mitigate potential biases from variations in data acquisition protocols with our preprocessing.

RC: ***More explanations are needed for the choice of time window. With the current description, it is not known how their network is constructed. I suggest the authors make an effort to develop the Sections, giving more notions on the network generation and the related deep learning algorithms.***

AR: We appreciate your inquiry regarding the choice of the 20-second window size. To address this concern, we complement the following paragraph in 2.1. Data and data preprocessing section:

"After the extraction of the BOLD time series, a sliding time window of 20 seconds was employed for data augmentation. This duration was selected based on our previous study [26], where it demonstrated optimal performance for the ASD dataset. Additionally, to ensure comparability between the ASD and ADHD datasets, the same window size was utilized for the ADHD dataset. By employing consistent time windows across both datasets, we aimed to mitigate potential biases arising from variations in data acquisition protocols between different sites and enhance the robustness of our analyses."

RC: ***Likewise, in the section Connectivity matrices, some necessary descriptions are missing.***

AR: We appreciate the opportunity to address your concerns and improve the clarity of our work. We have carefully considered your comments and made the following revisions to address the missing descriptions in the 2.2 Connectivity matrices section and provide additional details on the deep learning algorithms used. To address the missing descriptions of the deep learning algorithms utilized in our study, we have inserted Table 1 and Table 2, which present the architectural details and hyperparameter configurations for the Convolutional Neural Network (CNN) and Long Short-Term Memory (LSTM) models, respectively. These tables outline critical aspects of our deep learning approach, including layer configurations, activation functions, optimizer choices, and learning rates. Additionally, we have provided details about the dropout regularization technique employed in our models. We specify the dropout rates used in each layer to mitigate overfitting and enhance the generalization capabilities of our models. These revisions address your concerns and provide a more thorough description of our methodology. Further, if our work is published, we will make all the codes available on GitHub.

RC: ***This submission seems to be a mere application of machine learning and deep learning algorithms to ASD and ADHD, the authors should point out clearly the degree of methodological novelty of the current submission w.r.t. the previous work.***

AR: We appreciate the opportunity to address your concerns regarding the methodological novelty of our study in the context of previous work.

In response to your comments, we have substantially revised the manuscript to delineate the methodological advancements presented in our study compared to our previous work and existing literature.

Firstly, we emphasize that while our methodology does draw upon machine learning and deep learning algorithms applied to ASD and ADHD, the novelty of our study lies in several key aspects. Specifically, we have introduced a multiclass classification approach, departing from the binary classification explored in our prior work. This shift allows us to differentiate between individuals with ASD and ADHD more accurately and typically developing profiles, thus providing a more nuanced understanding of neurodevelopmental disorders.

Moreover, unlike previous studies that typically focus on utilizing correlation-based or network-based approaches independently, our study pioneers the simultaneous integration of both levels of data abstraction within a multiclass context. By incorporating complex network measures derived from correlation analysis, we offer a more comprehensive characterization of brain dynamics associated with ASD and ADHD.

Furthermore, to enhance the interpretability of our machine learning results, we have introduced the application of SHapley Additive ExPlanations (SHAP) values, a cutting-edge technique that identifies critical features within our model. This improves the interpretability of our findings and enhances the robustness of our classification results.

Additionally, we have incorporated three novel measures—Effective Information, determinism, and degeneracy coefficients—to analyze the segregation and integration concepts within brain networks. These measures provide a deeper understanding of the dynamic properties of brain networks in individuals with ASD, ADHD, and typically developing profiles, further advancing the field.

Importantly, to the best of our knowledge, this is the first study to employ the Shapley value methodology for a multiclass classification of ASD and ADHD. This innovative approach enhances the interpretability and robustness of our classification results, setting our study apart from previous research efforts.

Therefore, in the new version of the manuscript, we add subsection 1.3, which describes the research gap, subsection 1.4, which outlines our clear goals and hypothesis, and subsection 2.1, which explains the methodology's novelty.

RC: ***A brief comparison with the state-of-the-art can be included in the discussion section to highlight the impact of the study.***

AR: Thank you for your thoughtful feedback and suggestions on our manuscript. We appreciate the opportunity to improve the quality and impact of our study. We have carefully considered your comments and have made several revisions accordingly.

Regarding your suggestion to include a brief comparison with the state-of-the-art in the discussion section, we have taken significant steps to address this recommendation. Specifically, we have expanded the Introduction section to provide a more comprehensive overview of the current state-of-the-art research in the field. Additionally, we have incorporated a table (Table 1) to summarize the key findings and contributions of these relevant studies, thereby enhancing the clarity and coherence of our comparative analysis.

We believe these revisions strengthen the manuscript by providing readers with a clear understanding of how our study relates to and advances the current state of knowledge in the field. These additions will significantly enhance the impact and relevance of our research.

RC: ***The discussion was too sample, I think there should be more discussion about the physiological meaning of the results and why the current method achieved better classification performance.***

AR: Thank you for your thorough review of our manuscript and for providing valuable feedback on the 4.1 Connectivity matrices section. We have carefully considered your comments and have made significant revisions to address your concerns.

In response to your suggestion to provide a more in-depth discussion on the physiological implications of our results and the reasons behind the superior classification performance achieved by our method, we have included a new paragraph in the discussion section of the manuscript. This paragraph aims to elucidate the physiological significance of our findings and to provide insight into the factors contributing to the improved classification performance of our methodology.

We acknowledge the importance of discussing our results' physiological implications and have provided a comprehensive analysis in the revised discussion section. Specifically, we have highlighted the significance of network topology in characterizing brain data and have emphasized the superior accuracy of constructing connectivity matrices compared to conventional methods employing raw EEG data. Additionally, we have discussed the impact of employing distinct correlation metrics on detecting brain changes associated with various neurological disorders, underscoring the importance of selecting an appropriate correlation metric in achieving optimal performance.

The following paragraphs were added: "Overall, we obtained the best performance compared to the multiclass machine learning algorithm comparing ASD, ADHD, and TD in the literature, as described in the Introduction section. Table 4 concisely overviews the primary research using machine learning classification methods and the ASD and ADHD groups outlined in the Introduction section. Analysis from Table 4 reveals that our methodology outperforms existing multiclass approaches. In our prior research [29] focusing on EEG time series, we demonstrated the superior accuracy of constructing connectivity matrices compared to conventional methods employing raw EEG data.

Furthermore, in subsequent investigations [24, 31], we found that employing a distinct correlation metric yielded improved detection of brain changes associated with ASD and schizophrenia, respectively. Interestingly, TE proved effective in capturing such changes in the fMRI dataset. Thus, one of our hypotheses for achieving optimal performance revolves around selecting an appropriate correlation metric."

We believe that the inclusion of this new paragraph significantly enhances the discussion section of the manuscript and provides readers with a deeper understanding of the physiological relevance of our findings and the factors contributing to the superior classification performance of our methodology.

RC: ***Please discuss future directions regarding diagnostics and drug trials.***

AR: Thank you for your thorough review of our manuscript and for providing valuable feedback regarding discussing future directions regarding diagnostics and drug trials. We have carefully considered your comments and added the following paragraph to the 5. Conclusion section regarding our project, which we are currently developing:

"Additionally, we propose future work integrating our methodology with federated learning techniques as a promising avenue for advancing diagnostics and drug trials in neurodevelopmental conditions. Federated learning offers a solution to data privacy and scalability challenges inherent in large-scale neuroimaging and medical data studies [155, 156, 157]. This approach involves collaboratively training machine learning models across multiple institutions or datasets while preserving data decentralization, allowing for aggregating insights from diverse populations without compromising individual data security [158]. By harnessing the power of federated learning, we can enhance the robustness and generalizability of predictive models, enabling the development of tailored diagnostic tools for specific demographics or clinical settings and supporting the implementation of adaptive interventions that evolve alongside our understanding of neurodevelopmental

conditions [159, 160]. Integrating federated learning methodologies into our methodology and future investigations can accelerate the translation of research findings into clinical practice [161], thereby improving diagnostic accuracy and guiding personalized treatment strategies for individuals with ASD, ADHD, and other mental health conditions."

RC: ***Some English typos were noticed, the authors should carefully and thoroughly check the English writing to avoid these typos.***

AR: We appreciate your valuable feedback and constructive criticism. Regarding your observation about English typos, we sincerely apologize for any oversights in the manuscript. We assure you that we take the quality of our writing seriously and have carefully reviewed the paper multiple times to eliminate any errors. In light of your comment, we have conducted another thorough review of the manuscript and have made necessary corrections to address any remaining typos or grammatical errors. We believe these revisions have enhanced the clarity and readability of the manuscript.

2. Reviewer 2

First of all, this paper made three classifications on ASD, ADHD and TC, and carried out corresponding analysis on the two diseases. A good classification effect was achieved based on the used data, and the corresponding analysis was carried out. However, the paper still had the following shortcomings:

RC: ***The amount of data used is mentioned in the abstract, but it is not clear whether there are 40 subjects in three categories respectively or 40 subjects in three categories altogether.***

AR: We appreciate your attention to detail and the opportunity to clarify an important aspect of our study. Indeed, we apologize for any confusion regarding clarifying the dataset size in our abstract. To address your concern, we confirm that our dataset comprises 40 subjects in each of the three categories: Autism Spectrum Disorder (ASD), Attention Deficit Hyperactivity Disorder (ADHD), and typically developed (TD) controls, totaling 120 subjects in the study. We will ensure this information is accurately reflected in the revised abstract, providing clarity regarding the dataset size and composition.

RC: ***The amount of data used is too small, and the pre-processing part of the data should be detailed.***

AR: We appreciate the opportunity to address your concerns regarding our study's data size and preprocessing methods. To provide a more comprehensive understanding of our data preprocessing pipeline, we have revised the 2.1 Data and data preprocessing section of the manuscript to include detailed information on the preprocessing steps performed using the Neuroimaging Analysis Kit (NIAK) that we use in both data. Specifically, we have elaborated on the preprocessing steps, such as motion correction, slice timing correction, spatial normalization, and nuisance regression, ensuring transparency and reproducibility of our methodology.

Furthermore, we have incorporated additional details regarding the data augmentation techniques employed in our study. Specifically, we utilized data augmentation sliding window analysis to augment our dataset. Slicing window analysis involves segmenting the fMRI time series into windows, enabling us to capture temporal dynamics and enhance the robustness of our classification model. We have thoroughly explained the slicing window parameters used, including window size and the amount of the connectivity matrices amount used for the analysis, in the following paragraph:

"After the extraction of the BOLD time series, a sliding time window of 20 seconds was employed for data augmentation. This duration was selected based on our previous study [26], where it demonstrated optimal

performance for the ASD dataset. Additionally, to ensure comparability between the ASD and ADHD datasets, the same window size was utilized for the ADHD dataset. By employing consistent time windows across both datasets, we aimed to mitigate potential biases arising from variations in data acquisition protocols between different sites and enhance the robustness of our analyses. Through the data augmentation process, 600 matrices were randomly selected, ensuring an equal representation of each class"

RC: ***Lack of innovation, less workload, can appropriately increase the amount of data to carry out experiments.***

AR: We appreciate the opportunity to address your concerns regarding the methodological novelty of our study in the context of previous work.

In response to your comments, we have substantially revised the manuscript to delineate the methodological advancements presented in our study compared to our previous work and existing literature.

Firstly, we emphasize that while our methodology does draw upon machine learning and deep learning algorithms applied to ASD and ADHD, the novelty of our study lies in several key aspects. Specifically, we have introduced a multiclass classification approach, departing from the binary classification explored in our prior work. This shift allows us to differentiate between individuals with ASD and ADHD more accurately and TD profiles, thus providing a more nuanced understanding of neurodevelopmental disorders.

Moreover, unlike previous studies that typically focus on utilizing correlation-based or network-based approaches independently, our study pioneers the simultaneous integration of both levels of data abstraction within a multiclass context. By incorporating complex network measures derived from correlation analysis, we offer a more comprehensive characterization of brain dynamics associated with ASD and ADHD.

Furthermore, to enhance the interpretability of our machine learning results, we have introduced the application of SHapley Additive ExPlanations (SHAP) values, a cutting-edge technique that identifies critical features within our model. This improves the interpretability of our findings and enhances the robustness of our classification results.

Additionally, we have incorporated three novel measures—Effective Information, determinism, and degeneracy coefficients — not used in our previous analysis or used before to evaluate Brain changes due to ADHD and ASD to analyze the segregation and integration concepts within brain networks. These measures provide a deeper understanding of the dynamic properties of brain networks in individuals with ASD, ADHD, and typically developing profiles, further advancing the field.

Importantly, to the best of our knowledge, this is the first study to employ the Shapley value methodology for a multiclass classification of ASD and ADHD. This innovative approach enhances the interpretability and robustness of our classification results, setting our study apart from previous research efforts.

Therefore, in the new version of the manuscript, we add subsection 1.3, which describes the research gap, subsection 1.4, which outlines our clear goals and hypothesis, and subsection 2.1, which explains the methodology's novelty.

RC: ***LSTM has significantly better performance than SVM, so why not try LSTM for analysis instead of SVM for reducing computation?***

AR: Thank you for your insightful comments regarding choosing a machine-learning algorithm for our analysis. We appreciate your thorough review of our manuscript.

Regarding your suggestion to use LSTM instead of SVM due to its superior performance on the test set, LSTM showed promising results regarding accuracy metrics. However, we opted to use SVM for several

reasons, which we would like to clarify.

Firstly, while LSTM exhibited better performance on the test set than the train set, indicating potential overfitting, we implemented various regularization techniques, such as stratified cross-validation and dropout, to mitigate this issue. Despite these efforts, LSTM's superior performance on the test set could still be attributed to factors such as the architecture's complexity and the dataset's nature. As such, we proceeded cautiously and considered not only performance metrics but also computational efficiency in our choice of algorithm.

Secondly, SVM was chosen due to its widespread usage and effectiveness in similar classification tasks, as evidenced by its prevalence in the literature in the new manuscript version mentioned in Table 4. This familiarity with SVM among researchers facilitates comparison and reproducibility of our results with prior studies, enhancing the interpretability and generalizability of our findings.

Furthermore, considering the computational resources required for the subsequent analysis using the SHAP value methodology, we prioritized efficiency in our initial choice of algorithm. SVM offers a favorable balance between computational cost and performance, making it a practical choice for our study, particularly given the constraints of computational resources.

In summary, while we acknowledge LSTM's superior performance in our experimental setup, we believe that the choice of SVM for our analysis was justified based on computational efficiency, reproducibility, and the established effectiveness of SVM in similar contexts.

RC: ***In Figure 4-b, it is necessary to explain where many subjects come from and how to deal with them.***

AR: Thank you for your thorough review of our manuscript and for providing valuable feedback. Regarding your query about Figure 4-b, we acknowledge the need for further clarification on the data's origin and handling. As you correctly observed, the increased number of subjects depicted in the figure results from data augmentation through a sliding window approach. To address this concern more explicitly, we revised the relevant section of the methodology to provide a more precise explanation of how the sliding window approach was applied to augment the dataset. Specifically, we will include the rationale behind this approach, the parameters used for the sliding window, and how the augmented data was integrated into the analysis. Additionally, we enhanced the figure caption for Figure 4-b to explicitly state that the increased number of subjects results from data augmentation using the sliding window technique. This clarification will ensure that readers better understand the origin and processing of the data presented in the figure.

AR: The paper explains the use of transfer entropy to calculate the functional network, and the specific calculation formula can be written as much as possible in this place, and there is also a question that your connectivity matrix is symmetrical in Figure 1, whether it conforms to the connectivity matrix of transfer entropy calculation.

AR: Thank you for your insightful feedback on our manuscript. We appreciate your attention to detail and your valuable suggestions. We have carefully considered your comments and have made revisions accordingly. Below, we address each of your points:

1. **Explanation of Transfer Entropy Calculation Formula:** As you suggested, we have included the specific calculation formula for transfer entropy in the manuscript. This addition aims to provide clarity and transparency regarding the methodology used in our study.
2. **Symmetry of Connectivity Matrix:** In the previous manuscript version, we employed Spearman correlation to construct the connectivity matrix in Figure 1 to be aesthetically more harmonious.

However, we understand your concern regarding this approach's compatibility with the transfer entropy calculation. We apologize for the misunderstood. To address this issue, we have revised Figure 1 to reflect the use of normalized transfer entropy to construct the connectivity matrix. This modification ensures that the connectivity matrix aligns with the methodology described in the manuscript, thus maintaining consistency throughout our analysis.

We believe that these changes enhance the clarity and rigor of our study, and we are grateful for your assistance in improving the quality of our manuscript.
