# Supplementary material for: Multiclass classification of Autism Spectrum Disorder, attention deficit hyperactivity disorder, and typically developed individuals using fMRI functional connectivity analysis
Source: PLoS One. 2024 Oct 17;19(10):e0305630. doi: 10.1371/journal.pone.0305630 (PMC11486369; doi:10.1371/journal.pone.0305630)
Supplement: Attachment — Submitted filename: Author_response_to_review-version2.pdf [file pone.0305630.s004.pdf]

Authors’ Response to Reviews of

Multiclass machine learning and deep learning for analysis of fMRI functional connectivity from individuals with Autism Spectrum Disorder and Attention Deficit Hyperactivity Disorder

Caroline L. Alves1, Tiago Martinelli, Loriz Francisco Sallum, Francisco Aparecido Rodrigues, Thaise G. L. de O. Toutain, Joel Augusto Moura Porto, Christiane Thielemann, Patricia Maria de Carvalho Aguiar, Michael Moeckel
PLOS ONE,

RC: ***Reviewers’ Comment***, AR: Authors’ Response, ☐ Manuscript Text

We would like to thank you the reviewer for carefully reading our manuscript. The comments have improved the manuscript. We provide point-by-point responses to each comment below.

1. Reviewer 1

No Response.

2. Reviewer 2

RC: ***In the chapter 2.2 Data and data preprocessing, the spelling of "ABIDE" is missing "A"(page 5). You should pay attention to these spelling problems and check the words in the whole text as well as sentence problems.***

AR: Thank you for your thorough review and constructive feedback. We appreciate your attention to detail.

In Chapter 2.2, Data and Data Preprocessing, we have corrected the spelling of "ABIDE" by adding the missing "A" on page 5. Further, following your suggestion, we have carefully reviewed the entire manuscript for any additional spelling errors and sentence structure issues.
